# Socio-economic inequalities in minimum dietary diversity among Ethiopian children aged 6–23 months: a decomposition analysis

**DOI:** 10.3389/fpubh.2024.1422563

**Published:** 2025-01-29

**Authors:** Nigusu Worku, Amare Mesfin Workie, Lamrot Yohannes, Mihret Getnet, Wubshet Debebe Negash, Adina Yeshambel Belay, Lakew Asmare, Hiwot Tadesse Alemu, Demiss Mulatu Geberu, Melak Jejaw, Kaleb Assegid Demissie, Misganaw Guadie Tiruneh, Asebe Hagos

**Affiliations:** ^1^Department of Health Systems and Policy, Institute of Public Health, College of Medicine and Health Sciences, University of Gondar, Gondar, Ethiopia; ^2^Department of Nutrition and Dietetics, Institute of Public Health, College of Medicine and Health Science, University of Gondar, Gondar, Ethiopia; ^3^Department of Environmental and Occupational Health and Safety, Institute of Public Health, College of Medicine and Health Science, University of Gondar, Gondar, Ethiopia; ^4^Department of Human Physiology, School of Medicine, College of Medicine and Health Sciences, University of Gondar, Gondar, Ethiopia; ^5^Department of Epidemiology and Biostatistics, Institute of Public Health, College of Medicine and Health Sciences, University of Gondar, Gondar, Ethiopia; ^6^National Centre for Epidemiology and Population Health, The Australian National University, Canberra, ACT, Australia

**Keywords:** inequities, socioeconomic inequality, dietary diversity, concentration curve, concentration index, decomposition analysis, Ethiopia

## Abstract

**Background:**

Globally, inappropriate feeding practices account for more than two-thirds of all cases of child mortality during the first 2 years of a child’s life. For a long time, Ethiopia has suffered from the double burden of malnutrition: overnutrition and undernutrition both pose significant challenges, particularly for children. Undernutrition is mainly caused by wealth and educational disparities across the country. Therefore, this study aimed to assess the socio-economic inequalities in minimum dietary diversity (MDD) practices among Ethiopian children aged 6–23 months and the potential contributing factors.

**Methods:**

The study utilized the recent datasets of the Ethiopia Mini Demographic and Health Survey (EMDHS) of 2019. A total of 1,511 weighted samples were included in the research. Microsoft Excel and STATA v.14 software were employed to extract, clean, and analyze data. A probit model decomposition analysis was performed. The concentration index (CIX) and curve were used to examine household wealth status and maternal education level inequalities in the MDD.

**Results:**

The total weighted prevalence of MDD among children under the age of 5 in Ethiopia was 13.5%. Wealth and educational status show positive CIX values for inequality, as exhibited by the concentration curve under the equality line. The CIX with household and maternal education status were (CIX: 0.1034, *p* < 0.0029) and (CIX: 0.1057, *p* < 0.0002), respectively. This indicates a greater concentration of inequalities among privileged women. The decomposition analysis revealed that household wealth status, (58.23%) contributed by wealth status, (36.38%) place of delivery, (30.47%) maternal education, and (21.5%) administration region, explained the pro-rich inequalities in MDD in Ethiopia.

**Conclusion:**

This study identified significant inequalities in terms of wealth and maternal educational level in the context of MDD. Policymakers and healthcare workers should develop effective strategies to tackle the underlying causes of inequalities in attaining MDD, specifically focusing on household wealth and maternal education.

## Introduction

Malnutrition is a crucial public health challenge; more specifically, it results in numerous childhood health issues (1). Ensuring dietary diversity in child-feeding practice is essential for proper child growth and development (2). The Minimum Dietary Diversity (MDD) for Children is commonly used to evaluate the variety of foods consumed by children and breastfeeding practices (3). Malnutrition in children includes both the undernutrition type, such as wasting, stunting, and underweight, and the overweight type, such as obesity, which exposes the individual to different types of diseases, both communicable (pneumonia, measles, and diarrhea), and non-communicable (diabetes mellitus, heart ailments, and kidney diseases) (4, 5).

The “Convention on the Rights of the Child” states that a child has the right to receive a variety of food types (6). A 2022 report indicated that globally, approximately 148 million (22.3%) children are stunted—that is, too thin for their height—and 45 million (6.8%) are wasted—that is, too short for their height—despite many children receiving adequate nutrition and complementary food types (3). In South Asian countries, 23% (7) of the children received MDD; the figure for Burkina Faso was 18% (8). In Ethiopia, where 67% of the adult population suffered from stunting as children, the child nutrition budget is 55.5 billion Ethiopian birr annually—this is equivalent to 16.5% of the country’s GDP (9).

Childhood nutrition is a global concern and significantly impacts a country’s growth and development trajectory (10). Optimal nutrition constitutes the essential groundwork for realizing the rights of every child (11). Household food consumption and food access indices are measured based on dietary diversity (12). Addressing and eliminating malnutrition in Ethiopia is crucial for the growth and success of the health sector transformational plan (9). The government of Ethiopia has formulated a strategy to eliminate stunting among children under 2 years of age by 2030 (13). Initiatives to improve the nutritional status of children under 5 years of age are now included in the national food and nutrition policy and strategy document of Ethiopia. This document incorporates a working framework for the standardization of indicated nutrition interventions (14). Additionally, the health sector transformational plan provides a multi-sectoral coordination and performance management arrangement along with a multi-year implementation roadmap (15). However, the implementation of these policies and strategies in Ethiopia has not produced any significant improvement in childhood nutritional status. Moreover, the 6–23-month age group—a period of rapid physical and cognitive development—is critical in terms of growth and nutrition. During this period, children transition from exclusive breastfeeding to complementary feeding, which is essential for providing the additional nutrients required for their development. Adequate and diverse nutrition during this period is crucial to prevent malnutrition, stunted growth, and developmental delays and lays the foundation for a child’s lifelong health and well-being.

A few priority intervention areas indicated in the health policies and strategies as the determinant factors of MDD include the following: education, wealth status, employment, and media exposure of households (7). Antenatal care (ANC) visits (45%) and type of toilet facility (15%) are also important areas (2). Household wealth (29.8%) and educational status (11.8%) explain the pro-rich inequalities in MDD in Indonesia (16). There exists a minimum acceptable diet intake difference in urban (14%) and rural (10%) areas among children under 5 years of age in Ethiopia (17). Policy formulators should engage, and obtain feedback from, community members to promote more equitable access to all Reproductive, Maternal, Newborn, and Child Health interventions (18). Moreover, as per the researchers’ knowledge, there is no literature that can be accessed in Ethiopia to investigate socioeconomic inequalities in MDD among children. Therefore, this study aimed to assess the prevalence of MDD among children aged 6–23 months in Ethiopia and the contributing factors of inequalities.

## Methods

### Study design, area, and data-collection period

The 2019 Ethiopia Mini Demographic and Health Survey (EMDHS) dataset was utilized for this study (https://www.dhsprogram.com/data/dataset_admin/login_main.cfm website). Ethiopia, located at the Horn of Africa, lies between latitudes 3° and 15° North and longitudes 33° and 48° East, encompassing a total area of 1,100,000 square kilometers. It comprises 11 ethnically and politically autonomous regional states, along with two administrative cities. The data were collected through a community-based cross-sectional survey from 21 March to 28 June 2019.

#### Sampling procedure and study population

A two-stage stratified sampling design was employed. In the first stage, region-based stratification was performed. Next, the regions were stratified by rural and urban areas. Thereafter, using the probability proportional sampling technique, 94 urban and 211 rural enumeration areas were selected. In the second stage, applying a systematic sampling technique, households were selected from each enumeration area. Regarding child nutritional status, data were collected. In each selected household, women of reproductive age with children under 2 years of age were interviewed using an individual questionnaire after they were informed about the importance of the study, and ethical permission was obtained from each participant. The DHS database provides detailed information regarding the data collection and sampling procedures (19). The study population included children aged 6–23 months.

#### Outcome variable

MDD was the outcome variable. The outcome variable was coded as “yes” if the child aged 6–23 months received at least five out of eight groups of food types and as “no” if the child received four or fewer out of eight food groups during the previous days of the survey. The eight food groups were as follows: (1) breastfeeding, (2) flesh foods (poultry, fish, meat, and liver or organ meats), (3) eggs, (4) dairy products (cheese, yogurt, and milk), (5) tubers, roots, and grains, (6) nuts and legumes, (7) vegetables and fruits rich in vitamin A, and (8) other vegetables and fruits considering the World Health Organization (WHO) 2008 MDD classification approach (20).

### Contributing factors for socio-economic inequality of MDD

The following factors were used to conduct the decomposition of the concentration index (CIX): age of the mother (15–19, 20–34, 35–49), residence (urban, rural), age of the child in months (6–12, 13–18, and 19–23), respondent’s religion (Muslim, Orthodox, Protestant, and others), marital status of the respondent (single, married), sex of the household’s head (male, female), administrative region of Ethiopia (Established regions, city administration, and emerging regions), ANC follow-up (no ANC, < 4 visits, and ≥4 visits), place of delivery (home, facility delivery), birth order of the child (first, second, third, fourth, fifth, and sixth or above). The EMDHS calculated the wealth index of households based on the use of several indicator variables (17).

#### Socio-economic status

Two indicators were employed to assess the socio-economic inequality of MDD: the wealth index and educational status. The principal component analysis was applied to calculate the wealth index using household assets. Infrastructures and amenities included key household ownership assets (21). Household wealth status (highest, higher, middle, poorer, or poorest) and maternal educational status (no education, primary, or secondary and above) were measured as the main explanatory variables, as these variables were considered to calculate the CIX of MDD.

#### Data analysis

Primarily, data were filtered for the age range of 6–23 months, and thereafter, responses marked as “do not know” or missing were deleted. Finally, the figure of 1,518 unweighted children was obtained. Concentration curves were plotted to observe inequality in MDD based on the mother’s wealth status and educational level. The concentration curve of the 45° line represents equality; any deviation from this line indicates inequality. If the line of concentration curve is below 45°, it signifies that MDD was more concentrated among mothers who have good wealth status. Conversely, if it is above the 45° line, it suggests that MDD was concentrated among mothers with poor wealth status. The concentration curve interpretation for maternal education is the same as above. The CIX was calculated to quantify the level of concentration based on the formula developed by Jenkins, Kakwani, and Kakwani et al. (22–24).

The formula is as follows:


$$CIX=\frac{2}{\mu }cov\left(h,r\right)$$

The weighted mean of MDD is represented by μ and r represents the distribution of educational status and wealth among individuals; cov(h, r) denotes the covariance between h and r. The value of the CIX is from an interval +1 to −1. The value of the CIX closer to +1 implies the upper quantile of the variable higher concentration. Conversely, the value of the CIX closer to −1 implies the lower quantile of the variable higher the concentration. Finally, to observe the explanatory variables’ contribution to the CIX, a decomposition of the CIX regarding the explanatory variables was conducted. O’Donnell et al. have developed the decomposition analysis method (25).

The formula is as follows:


$$y=a\underset{k}{\sum }\mathrm{\beta xKx}+\varepsilon$$

Here, βk represents the coefficient of the k^th^ explanatory variable, Xk denotes the kth explanatory variable, and *ε* represents the random error term.

The CIX for MDD can be decomposed as a result of the above regression model, as follows:


$$CI=\underset{k}{\sum }\left(\frac{\mathrm{\beta kXk}}{\mu }\right)Ck+GCє/\mu$$

Where Xk is the average of the k^th^ explanatory variable, Ck is the concentration for the k^th^ explanatory variable, βkXk/*μ* represents the elasticity of dietary diversity for the k^th^ explanatory variable, GCє/μ represents the part of the CIX that cannot be explained by the explanatory variables, and all other notations hold their usual meanings.

After performing the decomposition, the percentage contribution of each variable was plotted. Additionally, the concentration of MDD was observed by calculating the concentration curve and index using the wealth status and educational level of the mothers. All *p*-values were compared at a significance level of 0.05.

## Results

### Background information

After data cleaning, 1,511 (weighted) children along with the background information of their parents were recruited. Approximately three-quarters (74.51%) of the parents were women aged 20–34 years, 44.56% of the women had no education, and 71.68% were rural dwellers. The result indicated that 51.81% of the children were male. A total of 39.05% of the children were in the age group of 6–12 months, 36.26% were in the age group of 13–18 months, and the remaining 24.69% were in the age group of 19–23 months (Table 1).

**Table 1:** Socioeconomic characteristics among children 6–23 months of age in Ethiopia, 2024.

Characteristics	Categories	Total *N* (%)	Having MDD *N* (%)
Age of children (months)	6–12	590 (39.05)	(136) 0.090267
	13–18	548 (36.26)	(256) 0.168956
	19–23	373 (24.69)	(234) 0.141589
Age of the mother (years)	15–19	111 (7.36)	(118) 0.077972
	20–34	1,126 (74.51)	(204) 0.135269
	35–49	274 (18.13)	(208) 0.137564
Education level of the mother	No education	673 (44.56)	(122) 0.080742
	Primary education	625 (41.38)	(235) 0.155837
	Secondary or higher	213 (14.06)	(333) 0.22053
Respondent’s religion	Muslim	555 (36.69)	(188) 0.124536
	Orthodox	498 (32.99)	(249) 0.164663
	Protestant	421 (27.86)	(170) 0.112769
	Others	37 (2.46)	(3) 0.001457
Marital status of the mother	Single	84 (5.55)	(492) 0.325614
	Married	1,427 (94.45)	(181) 0.12005
Sex of the household head	Male	1,302 (86.14)	(197) 0.130548
	Female	209 (13.86)	(207) 0.137189
Place of residence	Urban	428 (28.32)	(238) 0.157776
	Rural	1,083 (71.68)	(183) 0.121075
Administrative regions of Ethiopia	City administrations	138 (9.11)	(51) 0.033788
	Established regions	1,312 (86.80)	(205) 0.135553
	Emerging regions	62 (4.09)	(396) 0.262343
Antenatal care visits	No ANC visits	351 (23.99)	(176) 0.116472
	1–3 ANC visits	451 (30.84)	(180) 0.119054
	4 and above ANC visits	660 (45.17)	(235) 0.15584
Place of Delivery	Home	680 (45)	(141) 0.093329
	Health facility	831 (55)	(245) 0.162677
Birth order of the child	First	369 (24.43)	(231) 0.152595
	Second or third	547 (36.2)	(221) 0.146059
	Fourth or fifth	311 (20.59)	(182) 0.120409
	Sixth or above	284 (18.78)	(133) 0.087991
Wealth status of the household	Quintile 1 (poorest)	300 (19.87)	(95) 0.062848
	Quintile 2	318 (21.07)	(151) 0.099866
	Quintile 3	289 (19.13)	(221) 0.146356
	Quintile 4	265 (17.52)	(224) 0.148439
	Quintile 5 (richest)	339 (22.40)	(297) 0.196093

### Prevalence of MDD

The overall weighted sample size was 1,511 women who had children. The prevalence of MDD was 199 (13.15%). The prevalence of dietary diversity among children was 22% for mothers having higher educational status, 15.5% for mothers having a number of ANC visits totaling four or more, and 16.6% for mothers having health facilities deliveries (Table 1). Those variables were above the mean prevalence of MDD.

### Inequalities in minimum dietary diversity

**Figure 1:**
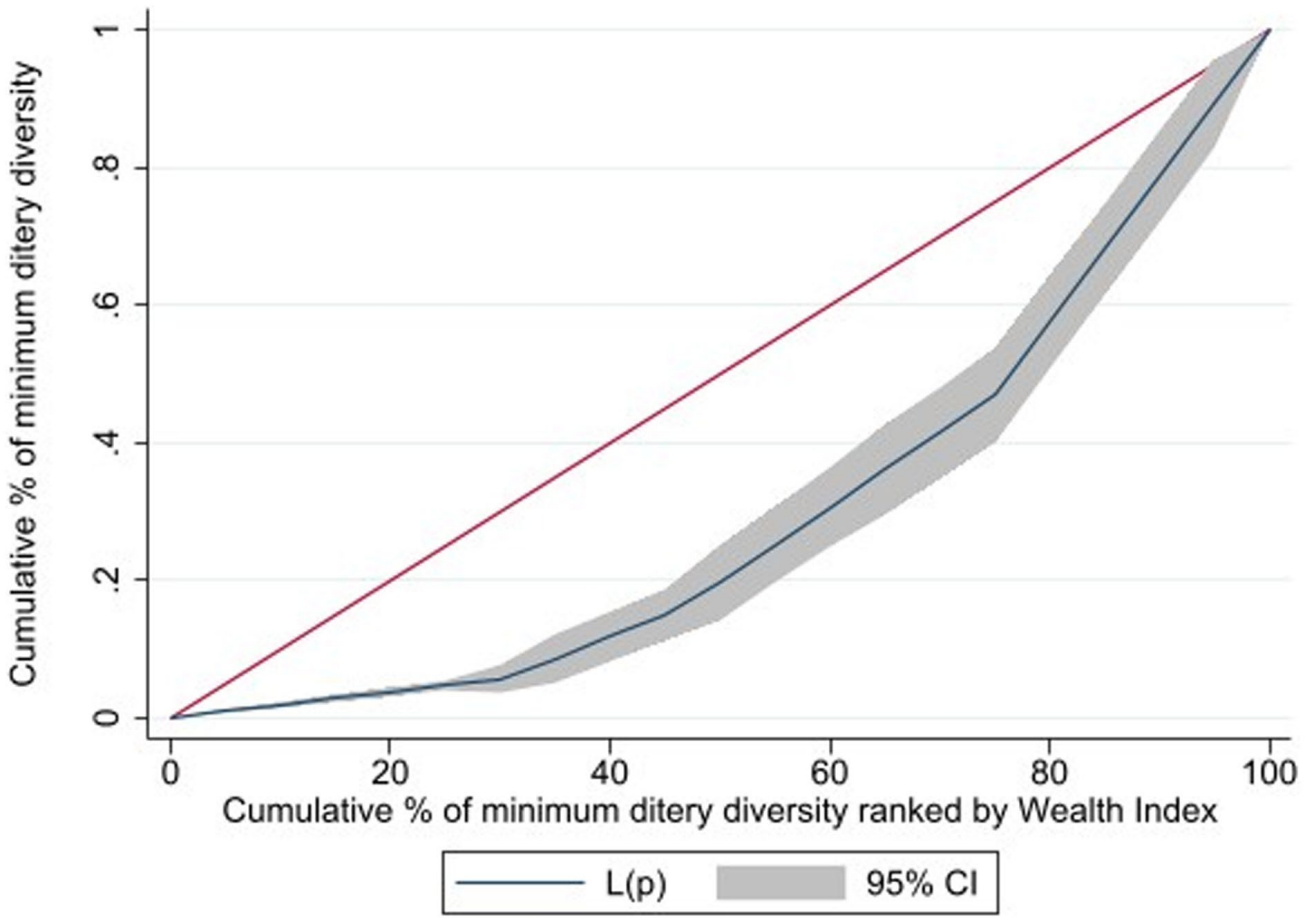
Cumulative percentage of minimum dietary diversity raked by wealth index children 6-23 months of age, in Ethiopia.

**Figure 2:**
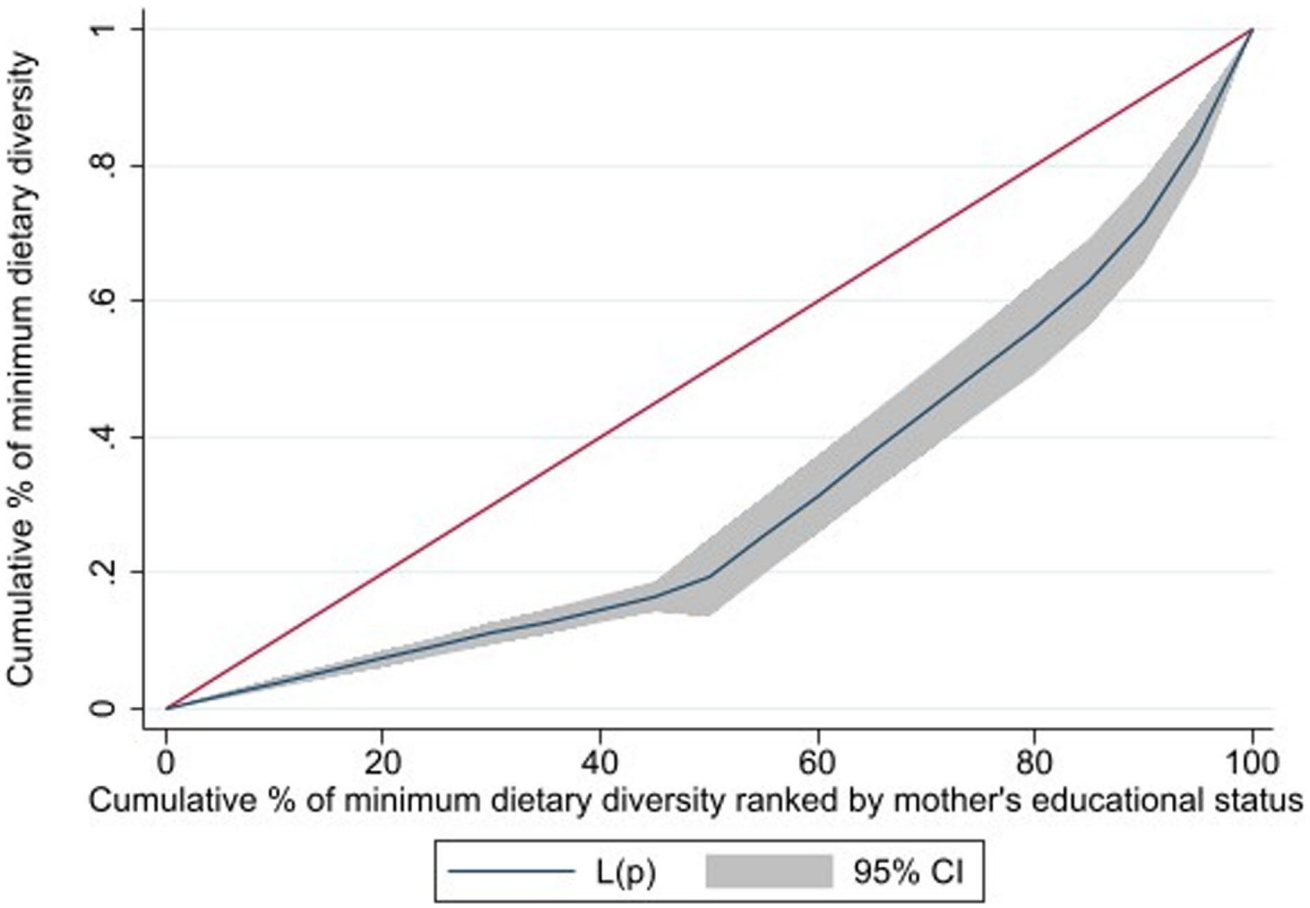
Cumulative percentage of minimum dietary diversity raked by maternal education status of children aged 6-23 months, in Ethiopia.

**Figure 3:**
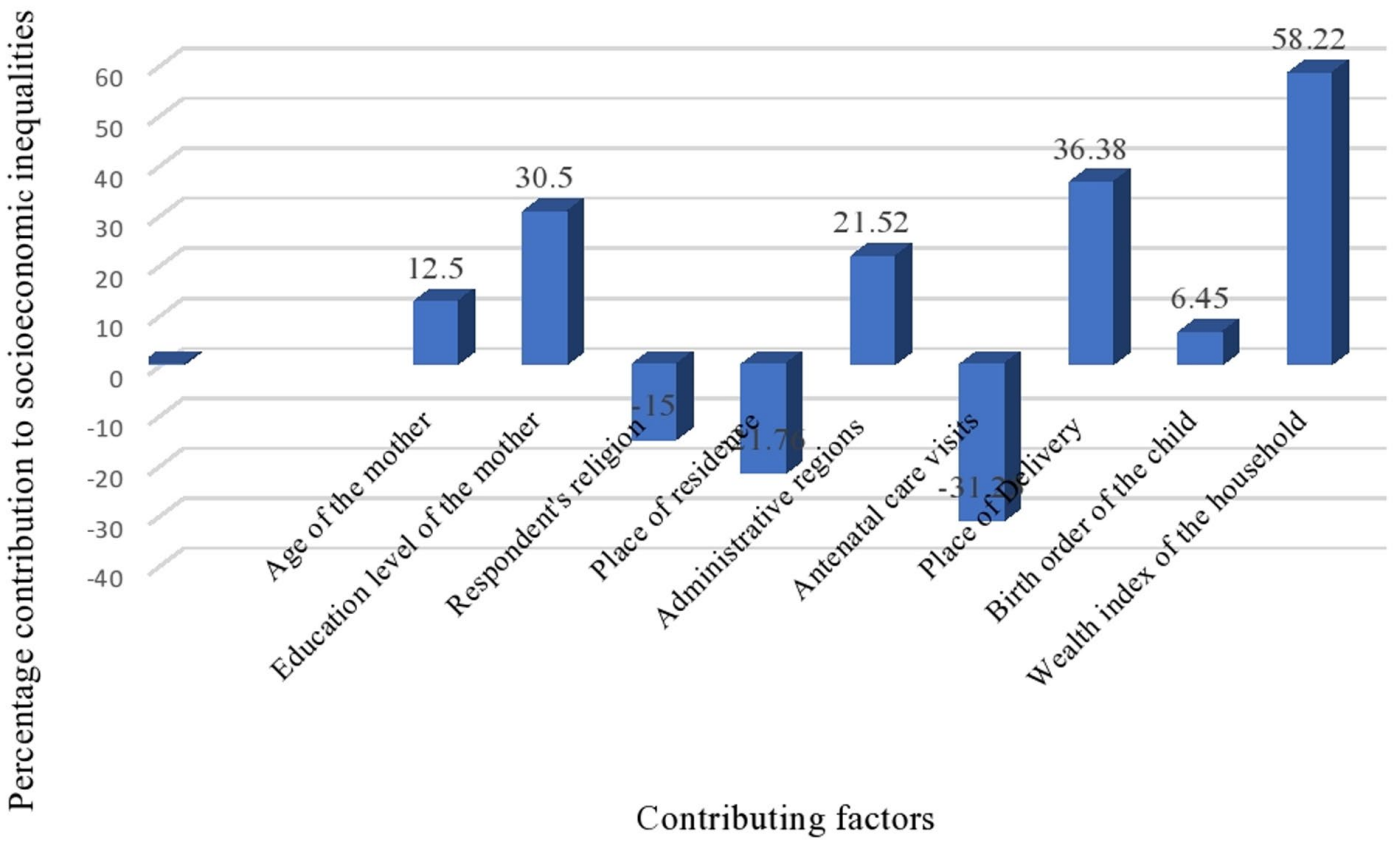
Percentage contribution of socio-economic inequality of MDD in Ethiopia.

From the concentration curve of MDD against the wealth status and maternal education, the concentration curve shows values below the 45° line. Thus, the MDD of children is more concentrated among the mothers with higher wealth status (Figure 1) and higher maternal education (Figure 2). The concentration indices by household wealth status and maternal education were significantly associated: (CIX: 0.1034, *p* < 0.0029) and (CIX: 0.1057, *p* < 0.0002) for MDD, respectively. Therefore, higher wealth and education status of the mothers were significantly concentrated with MDD (Figure 3).

### Decomposition of the concentration index

Decomposition of the CIX was conducted to observe the contribution of explanatory variables on inequalities. The column percentage contribution in Tables 2, 3 illustrates the relative contribution of each explanatory variable on the overall. A negative contribution indicates factor supports a decrease in concentration, and a positive contribution indicates factor supports an increase in the observed inequality. The wealth and educational levels decomposed with respect to the CIX were presented in Tables 2, 3, respectively. The decomposition analysis revealed the contribution of household wealth status to inequalities in MDD in Ethiopia: 58.23% contributed by wealth status, 36.38% by place of delivery, 30.47% by maternal educational level, and 21.5% by administration region. The contribution of ANC visits is −31.2% and place of residence −21.7%, which indicates that ANC visits and women’s place of residence supports a decrease of the observed CIX. Moreover, the observed CIX cannot explain 5.53% of the explanatory variables.

**Table 2:** A decomposition analysis of wealth status inequalities in MDD in Ethiopia, 2024.

Characteristics		Coefficient	Elasticity	Concentration Index	Contribution		
					Absolute contribution	Percentage contribution	Summed %
Age of children (months)	6–12	−0.04525	−0.07069	−0.02834	0.00200	1.9305	−0.327
	13–18	0.03694	0.05357	−0.04370	−0.00234	−2.258	
	19–23	Base	Base	Base	Base	Base	
Age of the mother (years)	15–19	Base	Base	Base	Base	Base	12.450
	20–34	0.07433	0.22155	0.07316	0.01620	15.65974	
	35–49	0.12179	0.08831	−0.03768	−0.00332	−3.20928	
Education level of the mother	No education	Base	Base	Base	Base	Base	30.497
	Primary education	0.07368	0.12195	0.11178	0.01363	13.17545	
	Secondary or higher	0.10298	0.05791	0.30944	0.01792	17.32238	
Respondent’s religion	Muslim	Base	Base	Base	Base	Base	−15.524
	Orthodox	−0.07378	−0.10829	0.12888	−0.01395	−13.4848	
	Protestant	−0.08589	−0.09571	0.03440	−0.00329	−3.18028	
	Others	−0.39626	−0.03897	−0.03042	0.00118	1.140648	
Marital status of the mother	Single	Base	Base	Base	Base	Base	−1.063
	Married	−0.18349	−0.069320	0.00159	−0.00110	−1.06332	
Sex of the household head	Male	0.03094	0.10661	−0.01049	−0.00111	−1.07298	−1.072
	Female	Base	Base	Base	Base	Base	
Place of residence	Urban	−0.03575	−04049	0.55621	−0.02252	−21.769	−21.769
	Rural	Base	Base	Base	Base	Base	
Administrative regions of Ethiopia	Established regions	0.13053	0.45322	0.04257	0.01929	18.64669	21.527
	City administrations	0.16358	0.02675	0.11173	0.00298	2.880619	
	Emerging regions	Base	Base	Base	Base	Base	
Antenatal care visits	No ANC visits	Base	Base	Base	Base	Base	−31.251
	1–3 ANC visits	−0.05571	−0.06872	0.00273	−0.00018	−0.174	
	4 and above ANC visits	−0.08972	−0.08972	0.35839	−0.03215	−31.0778	
Place of delivery	Home	Base	Base	Base	Base	Base	36.384
	Health facility	0.03298	0.07256	0.51885	0.03764	36.38473	
Child sex	Male	0.00433	0.00894	−0.00784	−0.00007	−0.06767	−0.067
	Female	Base	Base	Base	Base	Base	
Birth order of the child	First	0.02939	0.02872	0.17877	0.00513	4.958917	6.457
	Second or third	0.04048	0.05861	0.08449	0.00495	4.78492	
	Fourth or fifth	0.04007	0.03300	−0.10324	−0.00340	−3.28661	
	Sixth or above	Base	Base	Base	Base	Base	
Wealth status of the household	Quintile 1 (Poorest)	Base	Base	Base	Base	Base	58.22901
	Quintile 2	0.03972	0.03348	−0.33023	−0.01105	−10.6815	
	Quintile 3	0.08730	0.06679	0.00782	0.00052	0.502658	
	Quintile 4	0.07016	0.04918	0.26409	0.01298	12.54712	
	Quintile 5 (richest)	0.10877	0.09746	0.69530	0.05776	55.86073	
Explained						94.47	
Residual						5.53	

**Table 3:** A decomposition analysis of educational status inequalities in MDD in Ethiopia, 2024.

Characteristics		Coefficient	Elasticity	Concentration index (ECI)	Contribution		
					Absolute contribution	Percentage contribution	Summed %
Age of children (months)	6–12	−0.045	−0.070	−0.028	0.002	1.895	−0.32
	13–18	0.036	0.053	−0.043	−0.002	−2.215	
	19–23	Base	Base	Base	Base	Base	
Age of the mother (years)	15–19	Base	Base	Base	Base	Base	12.184
	20–34	0.074	0.221	0.073	0.016	15.332	
	35–49	0.121	0.088	−0.037	−0.003	−3.148	
Education level of the mother	No education	Base	Base	Base	Base	Base	29.846
	Primary education	0.073	0.121	0.111	0.013	12.894	
	Secondary or higher	0.102	0.057	0.309	0.017	16.952	
Respondent’s religion	Muslim	Base	Base	Base	Base	Base	−15.195
	Orthodox	−0.073	−0.108	0.128	−0.013	−13.201	
	Protestant	−0.085	−0.095	0.034	−0.003	−3.115	
	Others	−0.396	−0.038	−0.030	0.001	1.121	
Marital status of the mother	Single	Base	Base	Base	Base	Base	−1.043
	Married	−0.183	−0.693	0.001	−0.001	−1.043	
Sex of the household head	Male	0.030	0.106	−0.010	−0.001	−0.104	−0.104
	Female	Base	Base	Base	Base	Base	
Residence	Urban	Base	Base	Base	Base	Base	−21.30
	Rural	−0.035	−0.040	0.556	−0.022	−21.306	
Administrative regions of Ethiopia	Established regions	0.130	0.453	0.042	0.019	18.253	21.08
	City administrations	0.163	0.026	0.111	0.002	2.827	
	Emerging regions	Base	Base	Base	Base	Base	
Antenatal care visits	No ANC visits	Base	Base	Base	Base	Base	−30.594
	1–3 ANC visits	−0.055	−0.068	0.002	−0.001	−0.177	
	4 and above ANC visits	−0.049	−0.089	0.358	−0.032	−30.417	
Place of delivery	Home	Base	Base	Base	Base	Base	35.611
	Health facility	0.032	0.072	0.518	0.037	35.611	
Child sex	Male	Base	Base	Base	Base	Base	−0.066
	Female	0.004	0.008	−0.007	−0.001	−0.066	
Birth order of the child	First	0.029	0.028	0.178	0.005	4.856	6.318
	Second or third	0.040	0.058	0.084	0.004	4.684	
	Fourth or fifth	0.040	0.033	−0.103	−0.003	−3.222	
	Sixth or above	Base	Base	Base	Base	Base	
Wealth status of the household	Quintile 1 (poorest)	Base	Base	Base	Base	Base	56.645
	Quintile 2	0.039	0.033	−0.330	−0.011	−10.459	
	Quintile 3	0.087	0.066	0.007	0.001	0.494	
	Quintile 4	0.070	0.049	0.264	0.012	12.286	
	Quintile 5 (richest)	0.108	0.097	0.695	0.057	54.64522	
Explained						93.06	
Residual						6.94	

Similarly, the decomposition of the CIX was a product of the educational status of the mothers were (CIX: 0.1057, *p* < 0.0002) where 29.8% were contributed by maternal educational status, 56.64% by wealth status, 35.61% by place of delivery, and 21.1% by place of regional administration. Overall, these factors contributed to MDD inequality among children aged 6–23 months. The contribution by place of residence is −21.3% and − 30.5% by ANC visit, which indicates that place of residence and ANC visits help to decrease the observed CIX. Unexplained factors or residuals contributed to the remaining 6.94% of the inequality observed in this study.

## Discussion

This is a pioneering study to examine the wealth and educational status of the mothers and MDD inequalities among children aged 6–23 months in Ethiopia. It is also decomposed into the contributing factors. MDD prevalence was found to be 13.15% (199)—extremely low in comparison to other national studies (7, 26).

The results indicated that the WHO’s MDD criteria were met in the case of children from wealthier households and those born to mothers with higher educational attainments. The decomposition analysis revealed that 58.23, 36.38, 30.47, and 21.5% contributed by wealth status, place of delivery, maternal education, and region, respectively—this explains the pro-rich inequalities in MDD in Ethiopia. The result of pro-rich wealth-related inequalities in MDD was from previous studies (8, 16, 26–28).

The distribution of child MDD feeding practice was higher for women with higher educational status. The results are in line with those derived from research conducted in Pakistan, Indonesia, and India (2, 16, 29). The possible reason might be that educated women may be more aware of the importance of consuming a variety of food groups, their nutritional content, and the health benefits of dietary diversity. However, these findings do not imply the other way around; instead, they reveal that MDD is unfairly concentrated among children from the richest and most educated households (30).

Household wealth status is the principal factor for (58.23%) of inequalities in MDD—a finding in line with findings obtained in India and Bangladesh (27, 31, 32). Diet diversification depends on the accessibility, availability, and utilization of food groups; wealthier households are more likely to have enough and varied nutritious food groups or resources to consume (15). Therefore, high-income households are more likely to purchase (33) as well as enjoy greater access and availability of various food groups at the household level (34).

Similarly, the decomposition of the CIX computed based on the educational status of the mothers indicated that 29.8% was contributed by maternal educational status. Previous studies have suggested a direct influence of maternal education level on MDD practices for children (18, 35). This is probably because mothers with higher educational status possess better dietary knowledge, health literacy, dietary information-seeking behaviors and understanding, and critical thinking skills related to child nutrition. However, the contributions of the place of residence and ANC visits were − 21.3% and − 30.5, respectively, indicating that these factors help to decrease the observed CIX. Moreover, 6.94% of the observed CIX might not be explained. Initiatives and strategies focusing on enhancing women’s role in society, such as skill-based education, well-paid job opportunities, and better health facilities need to be implemented (2).

The decomposition analysis revealed that household wealth status, of which 36.38% was contributed by place of delivery and 21.5% administration region, explained the pro-rich inequalities in MDD in Ethiopia. Mothers who had delivered in a health institution had better access to information and counseling, received better follow-up care and guidance from healthcare providers, and supported breastfeeding practices. Therefore, it would be a positive contribution to MDD. Counseling by healthcare providers could contribute to improved diet diversification for children (36).

National–regional arrangements also significantly contributed to MDD among mothers with children aged 6–23 months. In the Ethiopian context, regional administration can be categorized as follows: emerging, established, and city administration. Emerging administration could experience challenges related to infrastructure, access to diverse food sources, and cultural and traditional feeding practices considered inappropriate (37, 38). Established and city administrations have relatively better access to a variety of food groups, infrastructure, health information, and health services, resulting in better MDD utilization. However, the contribution of ANC visits is −31.2% and that of place of residence is −21.7%, indicating that these factors had a role in decreasing the observed CIX. Additionally, 5.53% of the observed CIX was not explained by the contributor variables. The imparting of nutrition education to women to improve the practice of diversified diet in Ethiopia began with the Sekota declaration campaign. Despite the palpable improvement, the challenge persists.

### Strengths and limitations

This study utilized the latest dataset to assess the wealth- and education-related disparities in MDD in Ethiopia. The nuanced examination of the method of analysis (decomposition) helped ascertain the specific factors contributing to the overall outcome, and provided a quantifiable measure of the extent to which each factor contributes to the observed variation. The generalizability of the findings could be extended to the broader population of Ethiopia. Nonetheless, MDD is assessed based on a 24-h food recall, which could induce inaccuracy and recall bias. Although the analysis of decomposition helps to identify multiple elements influencing MDD inequality, causal links could not be established. This challenge also arises when working with cross-sectional data. To mitigate this limitation, we recommended longitudinal studies for future researchers.

## Conclusion

This study indicated significant disparities in the socio-economic inequality related to MDD among children aged 6–23 months in Ethiopia based on differences in the wealth and educational levels of households. The findings highlight the importance of using multi-sectoral strategies to tackle the root causes of socio-economic disparities in MDD. Therefore, policymakers and healthcare workers should prioritize women with low levels of income and education. Improving access to and quality of institutional maternal health services is essential to creating awareness regarding diet diversification for children. Nutrition-sensitive actions require multi-sectoral collaboration among the agriculture, health, and education sectors—among others—to address educational disparities and wealth inequality for significant improvement in MDD among children. By implementing targeted educational programs that promote nutritional awareness and providing financial support to low-income families, policymakers can ensure that all children have access to a diverse and nutritious diet. Additionally, integrating nutrition education into school curricula can empower families and communities to make informed dietary choices, ultimately contributing to better health outcomes for children.

## Data Availability

The original contributions presented in the study are included in the article/supplementary material, further inquiries can be directed to the corresponding author.
